# Association between quality of life and emotional overeating — a cross-sectional study in Danish children attending a multicomponent lifestyle camp

**DOI:** 10.1007/s00431-023-05206-7

**Published:** 2023-09-30

**Authors:** Ida Aagaard, Dorthe Dalstrup Jakobsen, Jens Meldgaard Bruun

**Affiliations:** 1 Steno Diabetes Center Aarhus, Aarhus University Hospital, Aarhus N, Denmark; 2 Department of Clinical Medicine, University of Aarhus, Aarhus N, Denmark; 3 Danish National Center for Obesity, Aarhus N, Denmark

**Keywords:** Quality of life, Emotional overeating, Eating behavior, Children and adolescents, Overweight and obesity

## Abstract

Emotional eating seems to emerge during the transition from childhood to adulthood; however, limited research has explored the association between emotional overeating and quality of life (QoL) in children with overweight and obesity. Therefore, the aim of this study was to examine the association between QoL and emotional overeating in a Danish sample of children with overweight and obesity. The present cross-sectional study is based on baseline questionnaire data from a nonrandomized controlled trial. Children attending a 10-week multicomponent lifestyle camp from October 2020 to March 2022 was invited to participate. Multiple linear regressions were used to examine if QoL was associated with emotional overeating before starting camp. In total, 229 children were included, and 45 children were excluded due to missing data, leaving 184 children in this study. The children had a mean age of 11.8 years (SD ± 1.38), with 60.9% girls and 39.1% boys, and the majority (94.6%) had overweight or obesity defined by a Body Mass Index Standard Deviation Score (BMI-SDS) > 1 SD. On average, children with a high tendency of emotional overeating had a 13.7 (95% CI 18.9; 8.5, *p* < 0.01) lower QoL score compared to children with a low tendency of emotional overeating.

*  Conclusions*: This study shows that children with a high tendency of emotional overeating have lower quality of life, compared to children with a lower tendency of emotional overeating. Due to study limitations, the findings should be supported by further research. (*Trial registration*: clinicaltrials.gov with ID: NCT04522921).

**Table:** 

**What is Known:** *• Emotional eating seems to emerge during the transition from childhood to adulthood.* *• Limited research has explored the association between quality of life and emotional overeating in children with overweight and obesity.*	
**What is New:** *• Children with a high tendency of emotional overeating had a lower quality of life compared to children with a lower tendency of emotional overeating.* *• Emotional overeating was negatively associated with quality of life in children with overweight and obesity.*

**What is Known:**

*• Emotional eating seems to emerge during the transition from childhood to adulthood.*

*• Limited research has explored the association between quality of life and emotional overeating in children with overweight and obesity.*

**What is New:**

*• Children with a high tendency of emotional overeating had a lower quality of life compared to children with a lower tendency of emotional overeating.*

*• Emotional overeating was negatively associated with quality of life in children with overweight and obesity.*

## Introduction

Quality of life (QoL) is a comprehensive and multidimensional construct including several domains of subjective experiences such as physical ability, psychological well-being, social interactions, and school performance [1]. In research, health-related QoL is often used to measure well-being, and according to the World Health Organization, QoL is defined as a person´s perception of his/her position in life within the context of the culture and value systems in which he/she lives and in relation to his/her goals, expectation, standards and concerns [2, 3]. It is widely recognized that children with overweight and obesity have an impaired health-related QoL compared to children with normal weight [4–6], and one previous study found that children with severe obesity had similar QoL as children diagnosed with cancer [4]. In addition, children with overweight and obesity suffer from symptoms of depression and anxiety, poor self-esteem, and social stigma which complicate childhood obesity management [6]. The prevalence of disordered eating and binge eating (overeating with loss-of-control eating) has also been associated with weight status among children and adolescents [7, 8]. A previous cross-sectional study demonstrated that 17% of 8–12-year-old children with overweight and obesity had clinically significant levels of disordered eating [8] and weight concerns seem to be a risk factor for disorder eating (i.e., purging and binge eating) in adolescents [9]. Moreover, several studies found that binge eating increases the risk of developing eating disorders, general maladjustment, and psychopathology, e.g., a higher use of maladaptive emotion regulation strategies [5, 8–10].

Emotional eating defined as eating as a response from negative or overwhelming feelings is highly prevalent in adults with obesity [11]; however, in children, the prevalence of emotional eating seems to be very low suggesting that emotional eating emerges in the transition between childhood and adulthood [12]. To our knowledge, no previous study has explored the association between QoL and emotional overeating in children with overweight and obesity, and exploring this may help identify modifiable behaviors which could exacerbate negative physical and psychosocial consequences of childhood obesity [8].

Therefore, the aim of the present study was to investigate whether there is an association between QoL and emotional overeating in a Danish sample of 7–14 years old children with overweight and obesity.

## Methods

The present cross-sectional study is based on baseline questionnaire data from the COPE-study, which is a nonrandomized controlled trial (clinicaltrials.gov: ID: NCT04522921). The complete study design has been reported in a different paper *(Under review).*

Briefly, participating children were recruited in collaboration with two well-established multicomponent lifestyle camps in Denmark. Children from 7-14 years of age, who struggle with overweight/obesity, low self-esteem, and/or unhappiness, could be referred to attend camp for 10 weeks. The lifestyle camps focus on a healthy lifestyle and aim to improve QoL in children. All children attending camp from October 2020 to March 2022 were invited to participate and parents/guardians provided written consent for their child to participate in this study.

## Measurements

Background characteristics, e.g., sex and household income per year, were assessed by a self-developed parent-reported questionnaire. Camp staff measured body weight (kg) and height within the first week of camp. Body weight (kg) was measured according to standard procedures using a bioelectric impedance (InBody model 270, Hopkins Medical Products, Grand Rapids, MI, USA) and height (meters) was measured using a fixed wall measuring tape. An age-and-sex-adjusted Body Mass Index Standard Deviation Score (BMI-SDS) was calculated using WHO AnthroPlus software, and children with a BMI-SDS > 1SD were defined as having overweight and children with a BMI-SDS > 2SD were defined as having obesity [13].

### The pediatric quality of life inventory questionnaire (PedsQL 4.0)

QoL was measured using the validated Danish translation of the Pediatric Quality of Life Inventory 4.0 questionnaire (PedsQL 4.0.), and permission to use the PedsQL questionnaire was granted by the original author [14]. PedsQL contains 23 questions subdivided into four dimensions: physical functioning, emotional functioning, social functioning, and school functioning. All questions are answered on a five-point Likert scale (0 = never a problem; 4 almost always a problem). Items are reverse scored and transformed to a 0–100 scale with higher scores indicating better QoL. The present study focus on the total QoL score.

### Child eating behavior questionnaire (CEBQ)

The original Child Eating Behavior Questionnaire (CEBQ) [15] was translated to Danish by two translators employed at a professional translation company (www.vidkom.dk). The full translation and validation process has been described in a different paper (*Under review*). Briefly, the translator translating the CEBQ from English to Danish was English native speaking while reverse translating was performed by an English native speaking. The translation process was summarized in a consensus report. A confirmatory factor analysis confirmed the original factor structure and showed adequate internal reliability (Cronbach’s alpha ≥ 0.70) as reported by other studies [15–17].

CEBQ contains 35 questions subdivided into eight different eating behaviors: food responsiveness, enjoyment of food, desire to drink, satiety responsiveness, slowness in eating, food fussiness, emotional undereating, and emotional overeating [15, 18]. Each of the questions was answered on a five-point Likert scale (1 = never; 5 = always) with five questions being reverse scored. The eight different eating behaviors were scored from 1 to 5 with higher scores indicating a higher tendency of the behavior, just as a lower score indicates a lower tendency of the behavior. The present study focus on and examine emotional overeating (EOE-score), which is characterized by an increased intake of food in response to negative emotions, such as anxiety and anger. Secondly, the present study includes and examines food responsiveness (FR-score), which reflects a tendency to eat as a response to internal and external stimuli [15].

## Statistical analysis

Children were excluded from the present study if they did not answer the PedsQL questionnaire and the CEBQ at baseline. Data from the CEBQ did not fulfill the assumption of linearity, and therefore, the EOE-score and the FR-score were categorized into three categories: “Low” (score ≤ 2), “Medium” (score > 2–3), and “High” (score > 3). Descriptive analysis was stratified by EOE-categories. Differences within groups were tested using the chi-squared test for all included categorical variables. Categorical variables are displayed as absolute numbers and percentages [*n* (%)].

Multiple linear regressions were applied to determine the association between children’s QoL and EOE. Potential associated factors were considered in the statistical analysis including gender, BMI-SDS, and socioeconomic status measured as household income per year.

Based on previous evidence suggesting an association between food addiction and EOE [12, 19], secondary multiple regression analyses were performed  to test for effect modification between the EOE- and FR-scores. The first analysis included children with a low/medium FR-score (FR-score ≤ 3), and the second analysis included children with a high FR-score (FR-score > 3). The calculated coefficients together with a Wald test were examined to determine whether FR were considered an effect modifier. All statistical analyses were performed in Stata/MP 17.0 (StataCorp LLC, USA) with standard 5% statistical significance.

## Results

**Fig. 1:**
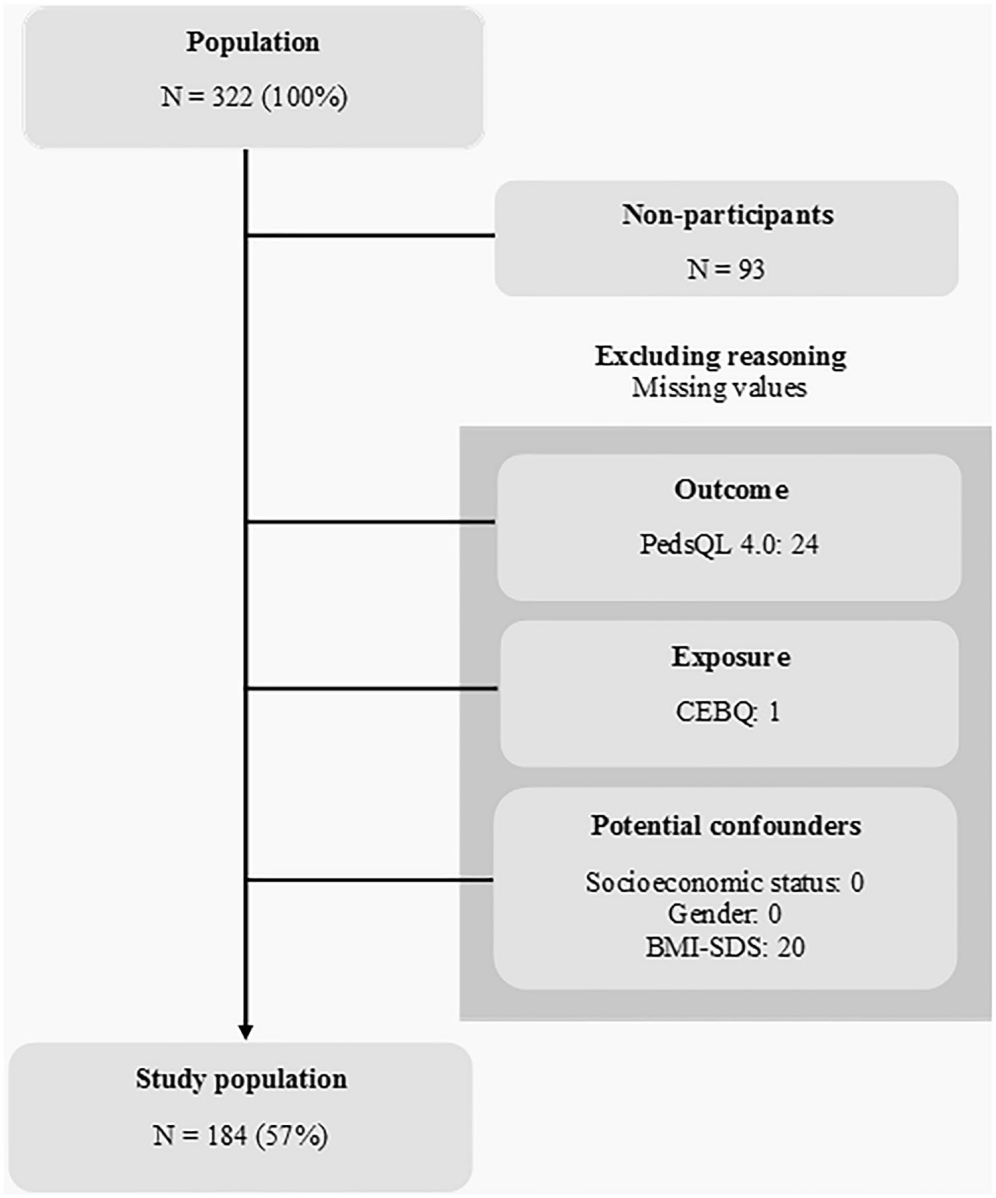
Flowchart of the study participants

In total, 322 children were invited to participate and 236 children accepted the invitation. Seven children withdrew from the study before or during camp, 93 children were non-participants, while 45 children were excluded due to missing data (Fig. 1).

Included children (*N* 184) had a mean age of 11.8 years (SD ± 1.38) with 60.9% girls and 39.1% boys. The majority of the children were categorized with a medium or high tendency to emotionally overeat (74.1%), and the average QoL score was 66.7 (SD ± 14.5). The majority of the children (94.6%) had overweight or obesity defined by a BMI-SDS > 1 SD, and 45.7% of the children were living in a household with an income between 200,000 and 499,000 DKK per year (Table 1). There were no significant differences in baseline characteristics between children categorized with a low, medium, and high EOE-score (Table 2).

**Table 1:** Participant characteristics (*N* 184)

**Gender**, *n* (%)	
Boy	72 (39.1)
Girl	112 (60.9)
**Age** (mean ± SD)	11.8 ± 1.38
**BMI-SDS**, *n* (%)	
≤ 1 SD	10 (5.4)
> 1– ≤ 2 SD	28 (15.2)
> 2 SD	146 (79.4)
**Household income (socioeconomic status)**, n (%)	
< 199,999 DKK per year	21 (11.4)
200,000–499,999 DKK per year	84 (45.7)
500,000–749,999 DKK per year	58 (31.5)
> 750,000 DKK per year	21 (11.4)
**EOE-score**, *n* (%)	
Low (≤ 2)	44 (23.9)
Medium (> 2–3)	71 (38.6)
High (> 3)	69 (37.5)
**Total QoL-score** (mean ± SD)	66.7 ± 14.5
**FR-score** (mean ± SD)	3.5 ± 1.0

**Table 2:** Participants characteristics divided by low EOE-score, medium EOE-score, and high EOE-score (*N* 184)

	**Low** **EOE-score**	**Medium EOE-score**	**High** **EOE-score**	***p*-value**
***N* (%)**	44 (23.9)	71 (38.6)	69 (37.5)	
**Gender, *n* (%)**				
Boy	19 (43.2)	26 (36.6)	27 (39.1)	
Girl	47 (56.8)	41 (63.4)	32 (60.9)	0.49
**BMI-SDS, *n* (%)**				
≤ 1 SD	4 (9.1)	3 (4.2)	3 (4.3)	
> 1– ≤ 2 SD	10 (22.7)	10 (14.1)	8 (11.6)	
> 2 SD	30 (68.2)	58 (81.7)	58 (84.1)	0.33
**Household income (socioeconomic status), *n* (%)**				
< 199,999 DKK per year	4 (9.1)	10 (14.1)	7 (10.2)	
200,000–499,999 DKK per year	17 (38.6)	32 (45.1)	35 (50.7)	
500,000–749,999 DKK per year	17 (38.6)	22 (31)	19 (27.5)	
> 750,000 DKK per year	6 (13.7)	7 (9.8)	8 (11.6)	0.81

Low EOE-score ≤ 2, medium EOE-score > 2–3, high EOE-score > 3

### Emotional overeating and quality of life

Multiple linear regression analysis revealed a significant negative association between QoL and EOE (Table 3, model 1). Compared to children with a low EOE-score, QoL was 6.5 points lower (95% CI 11.7; 1.3, *p* < 0.01) in children with a medium EOE-score and 13.7 points lower (95% CI 18.9; 8.5, *p* < 0.01) in children with a high EOE-score. These associations remained significant when adjusting for gender, BMI-SDS, and household income (Table 3, model 2).

**Table 3:** Correlations between the total QoL score and EOE-score (*N* 184)

	**Model 1**^**b**^	**Model 2**^**c**^
	**Coefficient (CI)**	**Coefficient (CI)**
Low EOE-score	Ref	Ref
Medium EOE-score	−6.5 (−11.7; −1.3)*	−5.5 (−10.6; −0.4)*
High EOE-score	−13.7 (−18.9; −8.5)*	−12,3 (−18.2; −7.9)*
Constant	74.4 (70.4; 78.5) < 0.001*	84.1 (77; 91.1)^**a**^ < 0.001*

Low EOE-score ≤ 2, medium EOE-score > 2–3, high EOE-score > 3

^*^p-value < 0.05

^a^The reference person is a boy with a normal weight, from a family with a household income between 200,000 and 499,999 DKK per year

^b^Model 1: unadjusted analysis

^c^Model 2: The analysis is adjusted for gender, BMI-SDS, and socioeconomic status

The secondary analysis (Table 4) showed that children with a high FR-score and a high EOE-score had a lower QoL compared to children with high FR-score and low/medium EOE-score (*p* < 0.01). However, the corresponding Wald-test showed a *p*-value of 0.33 = 33%, indicating that there was no effect modification between the two scores. Furthermore, due to the cross-sectional design of the study, the causality of the association cannot be determined.

**Table 4:** Effect modification between the FR-score and the EOE-score (*N* 184)

	**Coefficient**	**[95% conf. interval]**	***p*-value**
**FR-score: low/medium**			
Low/medium EOE-score	*Ref*	*-*	*-*
High EOE-score	−1.1	−12.2; 9.9	0.84
**FR-score: high**			
Low/medium EOE-score	*Ref*	-	-
High EOE-score	−9.6	−14.; −4.3	0.001*

Low/medium FR score/EOE-score ≤ 3; high FR-score/EOE score > 3

^*^p-value < 0.05

## Discussion

Based on the results of the present study, QoL is associated with EOE in 7-14-year old children. Children with a high tendency of EOE had a lower QoL compared to children with a lower tendency of EOE. However, due to the study design, it was not possible to determine causality.

The present findings are essential in line with current sparse evidence, suggesting that binge eating (overeating with loss of control) is a marker of QoL impairment [10, 20], also after adjusting for BMI [10]. In addition, the study by Ranzenhofer et al. [20] found that youth (12–17 years of age) with overweight and obesity reporting binge eating had significantly poorer health, mobility and self-esteem [20]. Low self-esteem, depression, body dissatisfaction, and weight and shape concern have been associated with the development of eating disorders [7], and, e.g., body dissatisfaction and concerns about shape and/or weight may be promoted by the combination of the thin-body ideal and an obesogenic environment [21]. A prospective study found that the association between EOE and BMI was bi-directionally across childhood and suggested that EOE was the only eating behavior trait possibly predicting weight gain, potentially leading to further body dissatisfaction and an increased risk of developing eating disorders [7, 22]. In the light of this vicious cycle, it seems that EOE is important to address in early childhood to improve QoL and prevent overweight and obesity in children and adolescents.

The rationale behind the present study was a lack of studies within this field in general and, in addition, a lack of studies investigating Danish children and adolescents, while the hypothesis was based on the existing literature in conjecturing a negative association between QoL and disordered eating [8]. The existing evidence, however, differ from the current study according to the choice of measuring instruments and target population, which complicates comparison of the results.

Current evidence investigating QoL and EOE in children and adolescents is scarce, and most evidence investigating this issue included adolescents and adults. Identified previous studies primarily examined the relationship between binge eating and QoL in adolescents and children [5, 8, 10]. Since the existing studies do not specifically focus on emotional overeating but rather on overeating in general, it raises the question of whether these findings can be directly compared to the results reported in the current study. Nevertheless, given the presence of negative correlations between QoL and overeating in general, it can be argued that a similar association might exist between QoL and emotional overeating [23]. Furthermore, a previous study investigating 13–17-year-olds and the association between food addiction and mental illness found a negative association between food addiction and mental illness [24]. Thus, the results of the present study show consistent similarities with the identified studies, which underlines that QoL and emotional overeating are important areas for future research.

## Strengths and limitations

Despite the novelty of the findings in the present study, some limitations are obvious. The representativeness of the target population is to some degree questionable as children attending camp has a slightly different social background (e.g., more children live with a single parent) and higher morbidity compared to children with overweight and obesity not attending camp [25]. However, more than 1000 children and adolescents are accepted for these camps annually and psychological outcomes (e.g., QoL and eating behavior) should be evaluated in this vulnerable group to plan proper interventions. The information collected depends on the questions asked and to whom they are asked, which means that other measuring instruments and other respondents might have given different results [26]. The questionnaires used in this study collected information about parts of everyday life often considered to be taboo, why it cannot be rejected that information bias exists in the data. Since the participation in this study is voluntary and anonymous, there is, however, no reason to believe that the study is tainted with serious differential misclassification and therefore information bias. In this study, the priority has been to present simple, rigorous models that are transparent and easy to convey. Therefore, the most relevant and meaningful confounders were selected based on the available literature, as well as accessible data [26, 27]. Lastly, its worth mentioning that due to the present study being an analytical cross-sectional study, it is not possible to determine the causality between the exposures and the outcome. The simple approach of the analyses made in the present study, however, can be useful to get an overall insight into this association. Furthermore, this study can contribute with new knowledge and serve as a basis for future research within the field, as only a limited number of Danish studies have explored the associations between QoL and EOE.

## Conclusion

Children who experience to emotionally overeat have deteriorated quality of life compared to children, who to a lesser extent tend to emotionally overeat. This association is not modified by the tendency to eat in response to internal and external stimuli (i.e., food responsiveness). However, since the present study was carried out as a cross-sectional study, future studies are warranted to investigate causality.

## Data Availability

The datasets used and/or analyzed during the current study are available from the corresponding author on reasonable request.
